# Using Stable Isotope Analysis to Understand the Migration and Trophic Ecology of Northeastern Pacific White Sharks (*Carcharodon carcharias*)

**DOI:** 10.1371/journal.pone.0030492

**Published:** 2012-02-15

**Authors:** Aaron B. Carlisle, Sora L. Kim, Brice X. Semmens, Daniel J. Madigan, Salvador J. Jorgensen, Christopher R. Perle, Scot D. Anderson, Taylor K. Chapple, Paul E. Kanive, Barbara A. Block

**Affiliations:** 1 Hopkins Marine Station of Stanford University, Pacific Grove, California, United States of America; 2 University of California Santa Cruz, Santa Cruz, California, United States of America; 3 Scripps Institute of Oceanography, University of California San Diego, San Diego, California, United States of America; 4 Monterey Bay Aquarium, Monterey, California, United States of America; 5 Point Reyes National Seashore, Inverness, California, United States of America; 6 University of California Davis, Davis, California, United States of America; 7 Max-Planck-Institut für Ornithologie, Radolfzell, Germany; 8 Montana State University, Bozeman, Montana, United States of America; Dalhousie University, Canada

## Abstract

The white shark (*Carcharodon carcharias*) is a wide-ranging apex predator in the northeastern Pacific (NEP). Electronic tagging has demonstrated that white sharks exhibit a regular migratory pattern, occurring at coastal sites during the late summer, autumn and early winter and moving offshore to oceanic habitats during the remainder of the year, although the purpose of these migrations remains unclear. The purpose of this study was to use stable isotope analysis (SIA) to provide insight into the trophic ecology and migratory behaviors of white sharks in the NEP. Between 2006 and 2009, 53 white sharks were biopsied in central California to obtain dermal and muscle tissues, which were analyzed for stable isotope values of carbon (δ^13^C) and nitrogen (δ^15^N). We developed a mixing model that directly incorporates movement data and tissue incorporation (turnover) rates to better estimate the relative importance of different focal areas to white shark diet and elucidate their migratory behavior. Mixing model results for muscle showed a relatively equal dietary contribution from coastal and offshore regions, indicating that white sharks forage in both areas. However, model results indicated that sharks foraged at a higher relative rate in coastal habitats. There was a negative relationship between shark length and muscle δ^13^C and δ^15^N values, which may indicate ontogenetic changes in habitat use related to onset of maturity. The isotopic composition of dermal tissue was consistent with a more rapid incorporation rate than muscle and may represent more recent foraging. Low offshore consumption rates suggest that it is unlikely that foraging is the primary purpose of the offshore migrations. These results demonstrate how SIA can provide insight into the trophic ecology and migratory behavior of marine predators, especially when coupled with electronic tagging data.

## Introduction

The white shark (*Carcharodon carcharias*) is a large apex predator that ranges across coastal and pelagic habitats in the northeastern Pacific (NEP) [1], [2], [3], [4]. White sharks are primarily concentrated along the west coast of North America during the late summer, autumn and early winter months and exhibit a regular offshore migration to oceanic habitats during the winter, spring and summer. Although some white sharks move as far as the Hawaiian island archipelago (traveling at least as far west as Midway Atoll), many sharks generally move to an area that has been referred to as the “offshore focal area” [2], “shared offshore foraging area” [3], or “white shark café” (hereafter referred to as the Café) [4]. This region is located approximately midway between Baja California, Mexico, and the Hawaiian Islands.

It has been theorized that the migratory behaviors elucidated by electronic tagging studies are related to foraging or reproduction [2], [3]; however the purpose of these offshore movements remains unclear. Recent electronic tagging studies have demonstrated that white sharks spend a considerable amount of their time in offshore habitats. Over the course of a year, sharks utilize pelagic habitats for 7 to 8 months or longer (mean 234 d±45 SD from pop-up archival transmitting (PAT) tags) [2], [4], indicating that these habitats play an important role in the life history of white sharks in the NEP. White sharks are listed as vulnerable by the World Conservation Union (IUCN) and protected under the Convention on the International Trade in Endangered Wild Flora and Fauna (CITES) [5]. Therefore it is important to understand how and why white sharks use these regions in order to have a more complete understanding of their life history and effectively manage and conserve the species.

To date, there has been no direct evidence of white sharks foraging during these offshore periods. It has been hypothesized that white sharks may be able to fast for extended periods of time following consumption of large quantities of high caloric marine mammal tissue [6]. It seems highly unlikely that an active, endothermic shark could fast for the extensive periods of time they spend offshore away from productive neritic habitats, suggesting that foraging in offshore habitats must occur. However, it has been noted that upon returning to aggregation sites off the central California coast (e.g., the Farallon Islands) after being offshore, white sharks often appear lean [7]. When in the Hawaii focal area, and to a lesser extent the Café, white sharks exhibit diel vertical migrations [4], suggesting that sharks may forage within the deep scattering layer, possibly upon mid-upper trophic level prey that track the deep scattering layer [8], [9], [10], [11]. White sharks in the Café, especially males, exhibit rapid oscillatory diving, a behavior which appears to be unique to this region and the purpose of which is unknown, but it has been theorized that it may represent searching or reproductive behavior [2].

The Café is an oligotrophic open ocean habitat in the North Pacific subtropical gyre where prey availability is presumably lower than in more productive neritic habitats [12]. Upon first examination, extensive use of this low-productivity region by a large apex predator is puzzling. However, there may be a more abundant forage base than apparent, as pelagic fishes (tuna, billfish) [13], [14], sharks [13], [14], and squid [15], [16], [17] occur in this region when white sharks are present. In addition, it is possible that shoaling of the oxygen minimum layer in the vicinity of the Café may aggregate prey by compressing vertical habitat [18], [19].

The Hawaiian Islands are a more productive region than the Café [20] with a diversity of pelagic, shelf and slope habitats that may offer white sharks more diverse and abundant prey. When in the coastal waters of Hawaii, white sharks may consume neritic teleosts, elasmobranchs, and cephalopods [21], [22], [23], [24]. In addition, Weng et al. [25] noted that white sharks have been observed near spinner dolphin (*Stenella longirostris*) aggregations and Hawaiian monk seal (*Monachus schauinslandi*) colonies, and that the timing of their occurrence in Hawaiian waters coincided with calving season of humpback whales (*Megaptera novaeangliae*) [26], suggesting that they may forage upon these species.

Although advances in electronic tag technology have provided great insight into the movements and habitat use of white sharks in the NEP, additional analytical techniques are required to help resolve some of the unanswered questions regarding the ecology of white sharks during the offshore component of their migratory cycle. SIA is a biogeochemical technique that uses the ratio of heavy to light isotopes of elements present in tissues as an intrinsic marker to understand the foraging and migration of an organism [27]. The stable isotope composition of an animal is directly related to that of its prey and the isotopic value generally shifts in a predictable manner through successive trophic levels [28], [29]. The stable isotope composition of an animal reflects that of local food webs [28] and because isotopic composition of different food webs vary spatially due to differences in biogeochemical processes, every animal is “tagged” [27], [30] and isotopes can be used as tracers to learn about trophic ecology and migration of organisms [30], [31], [32], [33].

Spatial differences in biogeochemical processes related to primary production create isotopic gradients, or isoscapes [27], [29], [30], [34], which can be used to understand the general patterns of movements and trophic ecology of white sharks. There is a gradient in ^13^C/^12^C ratios between nearshore or benthic derived food webs versus offshore or pelagic food webs, with ^13^C/^12^C ratios increasing from oligotrophic offshore to productive nearshore ecosystems [35], [36], [37], [38]. The nitrogen isotope composition at the base of the food web varies based on the ^15^N/^14^N ratios of nutrient sources (e.g., N_2_, nitrate, ammonium) and differential importance of biological processes such as denitrification, N_2_-fixation, and isotopic fractionation associated with nitrogen assimilation dynamics [39], [40]. These patterns generally result in offshore oligotrophic regions of the NEP, (i.e., subtropical gyre) being depleted in ^15^N due to the predominance of N_2_-fixation and nearshore regions of the California Current being enriched from the upwelling of ^15^N-enriched nitrate.

Because it directly reflects diet integrated over time and space, SIA is a useful complement to electronic tagging data, and an integrative approach combining both methods has the potential to be a useful tool that can be used to address a variety of ecological questions. White sharks move between isotopically distinct oceanographic regions during the course of their migrations. More productive coastal habitats are generally enriched in ^13^C and ^15^N relative to oligotrophic offshore habitats of the Café and Hawaiian Islands [29], [35], [39], [41], [42], [43], [44]. Because white sharks move between isotopically distinct oceanographic regions in a well-defined manner identified through electronic tagging studies, SIA has the potential to elucidate if sharks are foraging during offshore periods and improve our overall understanding of white shark trophic ecology. Indeed, because of the difficulty in studying white sharks due to their low population size, highly mobile nature, and protected status, SIA or other biochemical analyses of tissues collected non-destructively from sharks may be one of the few ways to understand trophic ecology and general patterns of habitat use of this species throughout its ontogeny and when they are away from visible, well-studied coastal regions.

The purpose of this study was to couple SIA and electronic tagging results from white sharks to provide insight into trophic ecology and migratory behavior of white sharks, a protected species in the NEP. By interpreting SIA results within the spatial and temporal context provided by electronic tag data, we demonstrate the efficacy of using this integrative approach to study the movements and trophic ecology of a large, highly migratory marine species. In particular, our goals were to directly incorporate electronic tagging data and isotopic incorporation rates into mixing models to 1) provide evidence of offshore foraging, 2) estimate relative importance of nearshore and offshore focal areas to white shark diet and 3) estimate relative consumption rates in the different focal areas.

## Materials and Methods

### Ethics statement

The project was conducted with permits from the California Department of Fish and Game, Monterey Bay National Marine Sanctuary, Gulf of the Farallones National Marine Sanctuary, U. S. National Park Service, Naval Postgraduate School, and under Stanford University animal care protocol 10765.

### Sample collection

A biopsy probe attached to a tagging pole was used to collect tissue samples from free-swimming white sharks during the tagging operations reported by Jorgensen et al. [4] at shark aggregation sites at South Farallon Island, Point Reyes, and Tomales Point in central California. Biopsies were from the epaxial musculature adjacent to the dorsal fin in the same region as tag implantation. Tissue was kept on ice until it was frozen several hours after collection, and stored at −80°C. Biopsies provided two tissue types, dermal and/or white muscle tissue. When both tissues were present, biopsies were subsampled for analysis of each tissue.

### Stable isotope analysis

Tissue samples were loaded in Accelerated Solvent Extractor (ASE, Dionex) cells between GF/F (Whatman), and sand was used to fill the remaining headspace. Each cell was rinsed 2× with Petroleum Ether (PE) and 3× with deionized water (DIW) to remove lipids and urea, respectively. Each rinse consisted of 9 ml of solution at 1500 psi and 50°C that was driven through samples for 5 min [45]. After PE and DIW treatment, ASE cells (containing filters and samples) were dried overnight in an oven set to 50°C. Following processing, 0.5–0.7 mg of dried tissue was weighed into tin boats (3×5 mm, Costech). Samples were analyzed at the Stable Isotope Laboratory at the University of California, Santa Cruz, using an Elemental Analyzer coupled to an isotope ratio monitoring mass spectrometer (Delta XP-EA, Thermo-Finnagen IRMS). Isotopic composition is expressed in δ values (parts per thousand differences from a standard or per mil (‰)) and is calculated using the equation: δ*X* = [(*R*_sample_/*R*_standard_)−1)] * 1000; where *X* = ^13^C or ^15^N, *R* = ratio of ^13^C/^12^C, ^15^N/^14^N, and the standards are Vienna Pee Dee Belemnite limestone (V-PDB) for carbon and AIR for nitrogen. Replicates of a gelatin standard within each analysis allowed for mass and drift corrections. Comparisons of this standard within and between runs yielded standard deviations of <0.1‰ and <0.2‰ for δ^13^C and δ ^15^N values, respectively.

### Isotopic characterization of focal areas

**Figure 1:**
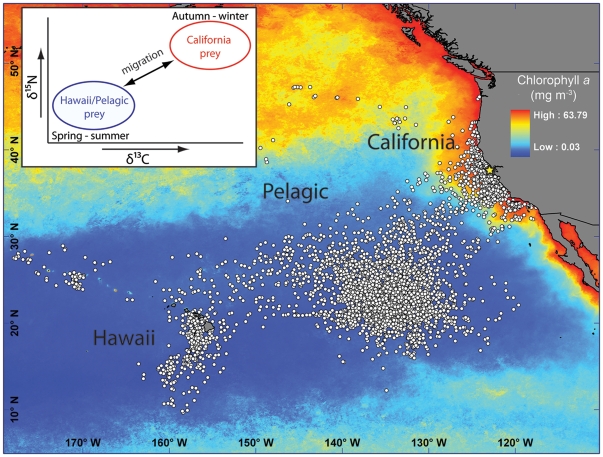
White shark focal areas from satellite tag data from Jorgensen et al. [**4**]. Regions used in the mixing model are indicated (California, Pelagic, Hawaii), see text for details. White shark aggregation sites in central California where tissue collection occurred are designated with the star. Mean chlorophyll-*a* concentration for 2006 (Aqua MODIS, http://oceanwatch.pfeg.noaa.gov) showing productivity gradients which isotopic gradients generally follow. Inset shows conceptual diagram showing how the seasonal migration of white sharks take them between regions with isotopically distinct prey.

Based on electronic tagging studies of central California white sharks [2], [4], we defined three general regions for white sharks: 1) the California Current, 2) offshore pelagic habitats of the subtropical gyre (i.e., the Café), and 3) neritic habitats of the Hawaiian Island Archipelago and pelagic habitats to the south of Hawaii. We refer to these regions as the California, Pelagic, and Hawaii regions (Figure 1).

The stable isotope values for all known and potential white shark prey in the NEP were collected from the literature, and prey were grouped according to the three defined regions in order to isotopically characterize the different focal areas (Table 1). Because marine mammals are believed to be the primary prey of large white sharks in central California [46], [47], [48], [49], we used marine mammal isotope values to estimate the California Current region value. Nothing is known about white shark diet in the Pelagic and Hawaiian focal areas. Therefore, we included all potential prey species from offshore habitats that have published stable isotope values. Potential prey items were defined as species that a white shark could reasonably be expected to encounter and consume either through active predation or scavenging and were from taxonomic groups known to be consumed by white sharks [50]. It is important to note that there are no published values of potential prey collected directly in the Café. However, the Café is part of the eastern subtropical gyre; thus species sampled from nearby regions of this large oceanographic region that have similar oceanographic conditions and biogeochemical processes [29], [30], [39], [42], [51] should be isotopically similar to those species in the Café. In addition, potential prey of white sharks in offshore habitats includes species that are not residents of the Café or Hawaii but occur seasonally in these regions and therefore might be encountered by white sharks in offshore habitats. As a result, stable isotope values from organisms sampled in different regions of the NEP were used to define the Pelagic region, which generally covers offshore, pelagic habitats between Hawaii and California, and serves as our proxy for the Café and similar habitats.

**Table 1:** Isotopic composition of potential white shark prey from different regions of the NEP.

		Carbon		Nitrogen						
ID	Species	Mean	SD	Mean	SD	Lipids	Region	Tissue	N	Reference
1	Grey whale (*Eschrichtius robustus*)	−13.1/−15.3	0.8	14.2	0.7	E	California	Bone collagen	13	[120]
2	Humpback whale (*Megaptera novaeangliae*)	−16.3	1.1	14.7	1.1	E	California	Skin	128	[121]
3	porpoise (*Phoecena phoecena*)	−16.2	0.5	15.2	0.8	E	California	Muscle	29	[122]
4	California sea lion (*Zalophus californianus*)	−13.8/−16.0	0.9	18.6	1.0	E	California	Bone collagen	15	[123]
5	elephant seal (*Mirounga angustirostris*)	−14.0/−16.2	1.0	18.2	1.0	E	California	Bone collagen	24	[123]
6	harbor seal (*Phoca vitulina*)	−12.4/−14.6	0.8	18.6	0.9	E	California	Bone collagen	18	[123]
7	northern fur seal (*Callorhinus ursinus*)	−14.5/−16.7	0.4	18.4	0.4	E	California	Bone collagen	6	[124]
8	Sperm whale (*Physeter macrocephalus*)	−13.3/−15.5	0.5	17.5	0.5	E	California	Tooth dentin	24	[120]
9	steller sea lion (*Eumetopias jubatus*)	−15.2	0.5	19.8	0.6	E	California	Muscle	5	[125]
10	humpback whale (*Megaptera novaeangliae*)	−18.0	0.9	13.0	1.4	E	Hawaii	Skin	310	[126]
11	yellowfin tuna (*Thunnus albacares*)	−16.5	0.4	10.2	1.8	E	Hawaii	Muscle	84	[127]
12	purpleback flying squid (*Sthenoteuthis oualaniensis*)	−17.8	0.3	12.8	1.0	C	Hawaii	Muscle	11	McCauley unpub. data
13	bigeye tuna (*Thunnus obesus*)	−16.3	0.4	11.3	0.9	E	Hawaii	Muscle	37	[128]
14	purpleback flying squid (*Sthenoteuthis oualaniensis*)	−18.7	0.5	8.1	1.1	N	Pelagic	Muscle	156,14*	[129], [130]
15	blue shark (*Prionace glauca*)	−17.6	0.6	16.8	0.8	E	Pelagic	Muscle	20	Leaf unpub. data
16	neon flying squid (*Ommastrephes bartrami*)	−18.4	0.7	11.7	1.3	E	Pelagic	Whole animal	44	[131]
17	Pacific pomfret (*Brama japonica*)	−19.2	0.8	10.9	2.1	E	Pelagic	Whole animal	10	[131]
18	northern right whale dolphin (*Lissodelphis borealis*)	−18.7	0.2	11.8	1.9	E	Pelagic	Unknown	23	[131]
19	bluefin tuna (*Thunnus orientalis*)	−18.2	0.3	12.6	0.8	C	Pelagic	Muscle	32	Madigan unpub. data
20	albacore tuna (*Thunnus alalunga*)	−19.2	0.4	13.4	0.6	C	Pelagic	Muscle	14	Madigan unpub. data

**Figure 2:**
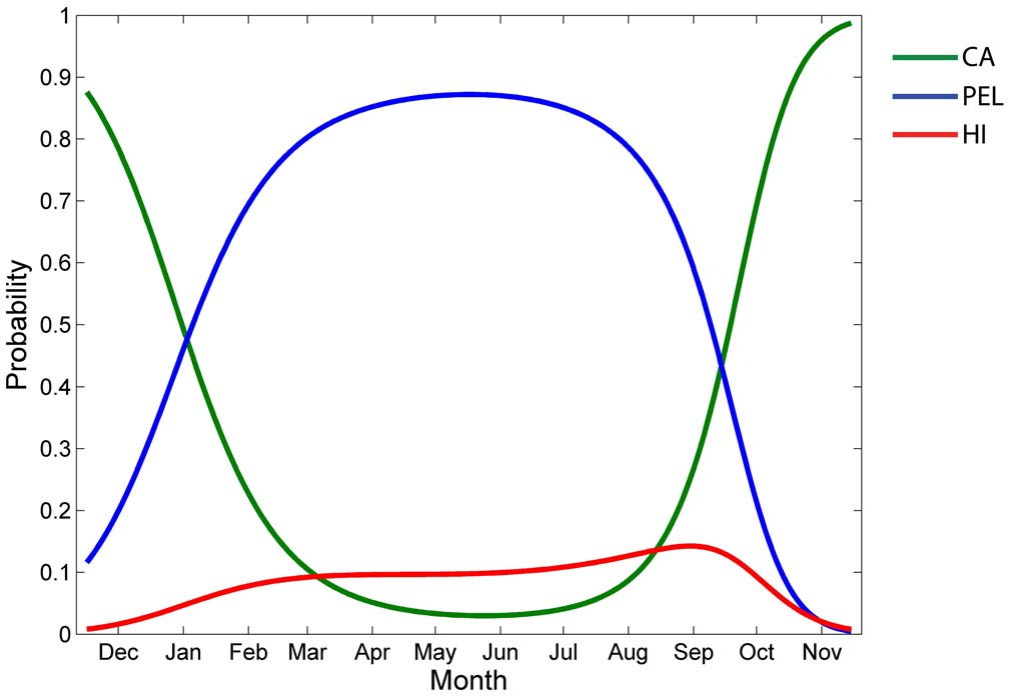
Probability of white sharks occurring in different focal areas by time of year based on satellite tag data from Jorgensen et al. [**4**].

‘ID’ is the number used to identify this species on Figure 2. For species with values based on collagen, both the reported values and values corrected to reflect muscle are reported respectively. ‘Lipids’ indicates if and how lipids were accounted for in the study (E = extracted, C = mathematically corrected, N: not accounted for), ‘Region’ denotes which focal area the prey are from and ‘N’ is sample size.

*156 squid used in estimating nitrogen, 14 in carbon.

All but one of the studies used lipid extraction or mathematical corrections to account for lipids in the tissues. For studies that measured collagen isotope values (bone and tooth dentin) in marine mammals, we adjusted collagen δ^13^C values to resemble muscle values by subtracting 2.2‰ [52]. Nitrogen was not adjusted because there is little variation in nitrogen fractionation between muscle and collagen in mammals [52], [53].

We calculated average δ^13^C and δ^15^N values for each of the three regions using a random effects meta-analysis in MetaWin version 2.0 [54], which has been used previously to synthesize stable isotope data [55]. Random effects meta-analysis weights species means by the inverse of sample variance and the between-sample variance [56]. These mean values were used as an estimate of generalized prey isotopic composition for the different regions. By including an array of potential prey items for each region, variability of mean estimates increased, which provided a more conservative estimate of regional values. See supporting information for more details about selection of prey used in the characterization of focal areas (Text S1).

### Stable isotope dynamics in white sharks

Two important biological parameters necessary to interpret stable isotope data are discrimination (or trophic enrichment) factors and tissue incorporation (turnover) rates. When an organism consumes a prey item, there is enrichment in ^15^N and ^13^C in the consumer's tissues relative to the prey due to preferential assimilation of the heavy isotope and preferential excretion of the light isotope [29], [31], [57], [58], [59]. This discrimination factor (Δ^13^C or Δ^15^N) is calculated as δX_consumer_ – δX_prey_ = Δ^13^C or Δ^15^N [58], [60], [61]. Studies often use a discrimination factor of 3.4‰ for nitrogen to estimate trophic level in elasmobranchs [62], [], [63,64], which is the average of terrestrial and aquatic organisms from Post [65]. However, elasmobranchs have unique physiological adaptations, such as urea retention for osmoregulation and lipid storage in the liver [62], [66], which may alter the fractionation processes during metabolism. We used discrimination factors from leopard sharks (*Triakis semifasciata*; 3.7‰±0.4 SD for nitrogen, 1.7‰±0.5 SD for carbon) reported by Kim [67] because they represent the first rigorous, lab-defined discrimination factors for elasmobranchs.

Isotopic incorporation, also known as turnover, is the period of time for a consumer's tissue to isotopically reflect a new dietary source following a shift between isotopically distinct diets. A variety of factors, including body size, growth rate (synthesis of new tissue), and tissue replacement due to catabolic turnover [68], [69], [70], [71] can affect an organism's tissue incorporation rate. Rates vary among tissues, with more metabolically active tissues (e.g., liver, blood, etc.) having more rapid incorporation rates than less active tissues (e.g., muscle, bone, etc.) [58], [60], [61]. The change in isotope composition is most commonly modeled by an exponential decay function based on exponential growth [58], [60], [71], [72], [73]. White shark tissue incorporation rates (λ) are unknown; therefore in our analyses we used three estimates of muscle incorporation rate that should bound white shark incorporation rate (Table 2). We used the tissue incorporation rate estimated for juvenile sandbar sharks [74] (Table 2), which were small, rapidly growing individuals [68]. Because incorporation rates are known to scale allometrically with body mass and the mass of white sharks is much greater than the mass of the juvenile sandbar sharks, the sandbar shark incorporation rate is likely faster than a large white shark's incorporation rate; thus, the sandbar shark rate was used as the upper limit of potential white shark incorporation rates. We also used two different relationships that scale incorporation rates allometrically with body mass (Table 2). White shark weights were estimated using the length-weight relationship from Compagno [50]. The mean weight of biopsied sharks was 781 kg±351 (SD). The allometrically scaled incorporation rate based on teleosts from Weidel et al. [75] was the slowest, lower limit of possible white shark incorporation rates. Although this relationship was based on small ectothermic fish (2.4×10^−8^ to 0.4 kg), muscle incorporation rates for leopard sharks (∼4 kg) were estimated within 90% confidence using this relationship [76]. We also used allometrically scaled incorporation rates from the equation in Carleton and Martinez del Rio [77] as a the intermediate rate as it was calculated for small endothermic birds (0.01 to 1.2 kg), which have relatively high incorporation rates, but being scaled for mass, this incorporation rate was slower than the sandbar shark rate. Both allometric equations describe carbon incorporation rate.

**Table 2:** Range of tissue incorporation rates used to approximate white shark incorporation rate.

Species	Incorporation rate (d^−1^)	Half life (d)	Reference
Sandbar shark	0.0060 (0.003)	129 (66)	[74]
Allometrically scaled bird	0.0028 (0.0001)	258 (46)	[77]
Allometrically scaled fish	0.0018 (0.0001)	394 (42)	[75]

All rates are for muscle tissue. Incorporation rate estimates are in days (d) and are reported as mean ± SE, whereas half life estimates are mean ± SD.

Tissue incorporation rate and metabolic activity are positively related in other taxa, including elasmobranchs, teleosts, crustaceans, birds and mammals [58], [60], [61], [78], [79]. White sharks are large active, endothermic sharks with increased metabolic rates [6], [80], [81], [82], hence they likely have increased tissue incorporation rates relative to other ectothermic elasmobranchs. Given their increased metabolic rate and active life style, white sharks likely have a incorporation rate between the allometrically scaled bird incorporation rate, which may be too rapid given the high metabolic rate of small endothermic birds, and the allometrically scaled fish incorporation rate, which is likely too slow due to the slow metabolic rate of small ectothermic fish. Although our extrapolation is beyond the size range of the studied species in these allometric relationships, we used three incorporation rates that should encompass the actual white shark incorporation rate for our analyses.

### Mixing model

Regions with different oceanographic and biogeochemical processes (i.e. coastal areas, upwelling currents, oligotrophic gyres) have different isotopic dynamics at the primary producer level. These differences propagate up the regional food webs and thus an oceanographic region can be represented by the isotopic values of prey species that occur in that region. If aggregate prey estimates differ sufficiently between regions, relative inputs of prey from different regions to a predator's diet can be estimated using Bayesian mixing models (i.e. MixSIR [83]). This approach is especially relevant to animals that show predictable migratory movements and in high trophic level organisms where regional baseline isotopic values may be difficult to identify and interpret after multiple trophic transfers (i.e., NEP white sharks). In addition, there is evidence that the stable isotope composition of apex predators better reflects large scale gradients in isotopes than primary producers [84], as apex predators integrate temporal and spatial variability in the isotopic composition of primary producers and lower trophic level organisms. Therefore, as the white shark is a highly migratory apex predator, with potentially migratory prey species, we chose to use mean prey values for the different focal regions based on known and potential prey as sources in our mixing model rather than using baseline (i.e., particulate organic matter or plankton) derived isoscapes for which data are lacking and which may be obscured by trophic transfer, seasonality, and migration patterns of prey at multiple trophic levels.

Because sharks use the focal areas at different times, the known movement patterns and dynamics of isotopic incorporation into new tissues (turnover rate) should be considered when using mixing models to elucidate use of the different focal areas. Existing Bayesian mixing models assume that tissues are in steady-state with their dietary components, which is unlikely in large, migratory organisms with slow incorporation rates. Due to their large size, white sharks likely have a slow incorporation rate that results in non steady-state conditions with diet. Thus information regarding spatio-temporal distribution of white sharks should be directly included into mixing models to provide a more robust estimate of focal area use. Fortunately, recent studies have demonstrated the extensibility of Bayesian mixing models by accounting for individual consumer variability [85] and the influence of spatial habitat patterns on consumer diets [86].

In this paper we extend the Moore and Semmens [83] model to account for both the temporal patterns in the distribution of sharks across the three focal regions (California, Pelagic, Hawaii) and the isotopic incorporation rate of tissues. Our modeling approach integrates satellite tagging data, tissue isotope values, and assumptions about isotopic incorporation in white shark muscle to characterize the relative contribution of different focal areas to white shark tissues and the relative feeding rate of sharks in the different ocean regions. While the modeling approach is described in detail below, we have also provided the JAGS/WinBUGS code as a digital appendix (Text S2).

In the original Bayesian mixing model formulation of Moore and Semmens [83], the *n* isotope signatures of *i* white shark tissue samples (*Iso_n_,_i_*) are described by:
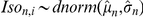


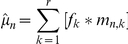


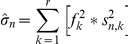
where *k* represents the number of sources considered and *f* represents the proportional contribution of each source to the mixture. In the above equations, *m* and *s^2^* represent the discrimination factor adjusted source isotope means and variances (i.e., source means and variances have been additively adjusted for discrimination factor means and variances).

We used satellite tagging data from 65 white sharks tagged as part of Jorgensen et al. [4] to estimate the proportion of the population residing in each of the focal areas as a function of Julian day of the year. The probability that a tagged shark *i* is in region *j* (1–3, for California, Pelagic, and Hawaii) on a given day *t* (1–365) is estimated by:
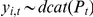
Where *y_i,t_* is the observed location of tagged sharks, and *P* is a matrix of region and time specific probabilities estimated from the 4^th^ order multinomial Logit function:
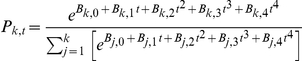
where all elements of *B_1_* are set to 0. As was noted in Jorgensen et al. [4], the probability of finding a white shark from the study population in coastal habitats was high during a relatively short period of time during the autumn and winter (October through December), while the probability of finding sharks in offshore habitats was greater between January and September (Figure 2). In our modeling framework, the *P* matrix is simultaneously treated as a daily estimate of the proportion of the population in each ocean region where, under a constant consumption rate across regions, we would expect the *f* vector (proportional contribution of each region to the shark isotope signatures) to be equivalent to the relative amount of time the population spends in each region (defined by the *P* matrix across all Julian dates). However, we are interested in determining whether there appears to be a differential contribution of each region to the diet of white sharks, beyond that expected from differences in the proportional occupancy of the shark population in each region though time. Therefore, we introduce region specific daily isotope uptake adjustment terms, *E*, to the elements of the *P* matrix:
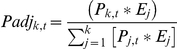
Here, we set the first element of the *E* vector (that applied to California) to 1 in order to make the isotope uptake adjustment of the other regions relative to the daily uptake rate in California.

Finally, because isotope values in white shark tissue “decay” at a rate defined by the isotopic incorporation rate, we expect that the contribution of a given day's foraging to white shark tissue isotopes will lessen the further into the future the sample is collected. Specifically, for a particular future day *t*, the fraction of original material remaining *C_k_* is:
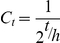
where *h* represents the tissue turnover rate (in half-lives) of stable isotopes (assumed to be the same for all stable isotopes). The *t/h* term thus represents the number of half-lives into the future that the sample was taken. Using this formula, we adjust the expected proportional contribution of each source:
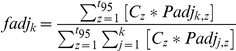
where *t_95_* is the number of days until 5% of less of isotope signature remains in the tissue. The modified mixing model was run for the three different estimates of incorporation rate.

## Results

A total of 53 white sharks (35 ♂, 7 ♀, 11 unknown) were biopsied from 2006–2009 (Table 3) from August to January. Most samples were collected from September to November (87%), with the highest number being collected during October (64%). Samples were primarily from male sharks. Sharks were sampled at Pt. Reyes (n = 15), South Farallon Island (n = 33), Tomales Point (n = 4), and Oregon (n = 1). All samples were from free-swimming sharks with the exception of the shark from Oregon, which was killed after becoming entangled in a crab pot. Sharks ranged in total length (TL) from 2.6 m to 5.3 m (mean ± standard deviation [SD] 4.3±0.6; hereafter all errors similarly expressed as SD). Based on an estimated TL at first maturity of 3.8 m for males [87] and 4.5 m for females [88], 31 sharks were mature, 11 were immature, and 11 were of unknown maturity (due to inability to identify gender of the shark).

**Table 3:** δ^13^C and δ ^15^N of white shark muscle and dermal tissue.

Tissue		N	Mean	SD	Min	Max
White muscle	δ^13^C	21	−15.5	0.5	−16.9	−14.4
	δ^15^N	21	18.4	1.0	17.4	21.1
	C∶N	21	3.3	0.1	3.2	3.5
Dermis	δ^13^C	48	−12.8	0.5	−14.1	−11.9
	δ^15^N	48	19.2	0.9	17.2	21.1
	C∶N	48	2.8	0.1	2.7	3.1

We analyzed 48 dermal (32 ♂, 6 ♀, 10 U) and 21 muscle (14 ♂, 2 ♀, 5 U) samples. Muscle and dermal tissues had distinct δ^15^N and δ^13^C values (Table 3). Sixteen biopsies contained both dermal and muscle tissue. For these paired samples, dermal tissue was significantly enriched in ^13^C and ^15^N relative to muscle (paired t-test, p<0.001) (Table 3). Because shark dermis is primarily collagen [89], mean dermal isotope values were corrected to resemble muscle according to tissue-specific differences reported by Hussey et al. [90] (corrected dermal values: −14.9‰±0.5 for δ^13^C and 21.5‰±0.9 for δ^15^N).

**Figure 3:**
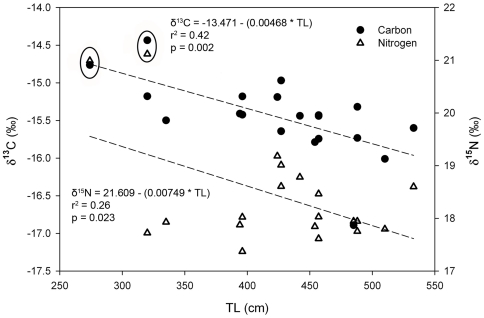
Relationship between white shark length (TL) and δ^13^C and δ^15^N of white shark muscle. The two ellipses contain the δ^13^C and δ^15^N values of two of the smallest sharks that had the highest δ^13^C and δ^15^N values.

There was a significant negative relationship between shark length and muscle δ^13^C values (linear regression, n = 20, p = 0.002, r^2^ = 0.42) and δ^15^N values (linear regression, n = 20, δ^15^N = 21.609−(0.00749 * TL), p = 0.023, r^2^ = 0.26) (Figure 3). Two of the smallest sharks (2.7 and 3.2 m TL) had the highest muscle δ^13^C and δ^15^N values of all sharks (−14.8‰, 21.0‰ and −14.4‰, 21.1‰ respectively). There was no significant relationship between dermal δ^13^C values and length (linear regression, n = 47, p = 0.368, r^2^ = 0.02). There was a significant negative relationship between dermal δ^15^N values and length (linear regression, n = 47, p = 0.048, r^2^ = 0.08), but the low r^2^ indicates that this relationship is very weak. As with muscle, some of the smallest sharks (2.6, 3.2, 3.3, and 3.4 m) had the highest δ^15^N values (20.4‰ to 21.1‰) of all the sharks. Of the muscle samples, 14 were from males, 2 from females, and 5 from sharks of undetermined sex.

**Figure 4:**
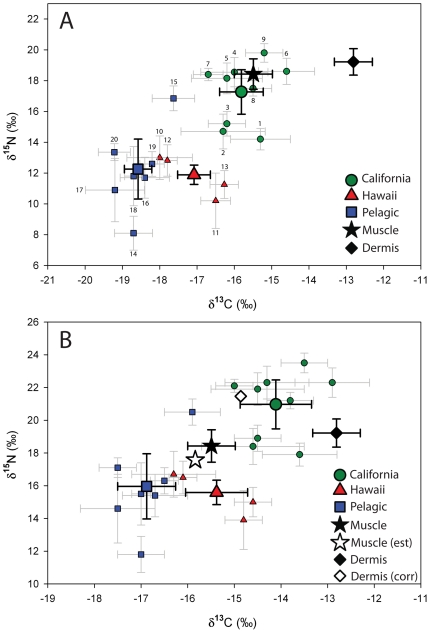
δ^13^C and δ^15^N values of white shark tissues and potential prey items from different focal areas. Regions and shark tissues are designated with different symbols and colors. For regions, smaller symbols show mean (SD) isotopic values of individual species and larger symbols indicating mean regional average (SD). 4a: δ^13^C and δ^15^N values for different prey, regions and white sharks tissues. Individual species are labeled according to Table 1. 4b: Mixing polygon used to estimate contribution of different regions to white shark tissue. Regional (and prey) values are adjusted to account for discrimination factor (Kim et al. [67]). Error in discrimination factors was propagated into error of mean regional values. Muscle (est) is the predicted mean stable isotope composition of white shark muscle if sharks foraged at the same rate in the different regions. Dermis (corr) is the mean dermal stable isotope composition adjusted to resemble muscle by accounting for differences in discrimination factors between the tissues; the adjustment is based on Hussey et al. [90]. Note that the closer the tissue values are to source (region) values, the higher the contribution of that source to the tissue.

Isotopic values of potential prey clustered by region (Figure 4, Table 4). The mean isotopic composition of prey from the California region was highly enriched in ^15^N and ^13^C relative to Pelagic and Hawaii prey. Hawaii prey were more enriched in ^13^C than Pelagic prey, but mean δ^15^N of Hawaii and Pelagic prey were similar, although the latter were more variable. The mean δ^13^C and δ^15^N values of all potential offshore prey items grouped together were −18.0‰±0.8 and 12.2‰±2.0, respectively. Muscle δ^13^C and δ^15^N values, adjusted to account for discrimination factor, were intermediate between California prey and offshore (Pelagic and Hawaii) prey (Figure 4b), demonstrating consumption of prey from offshore habitats. Dermis δ^13^C and δ^15^N values, adjusted to account for discrimination factor and corrected to resemble muscle tissue, was similar to California prey (Dermis (corr), Figure 4b), indicating coastal foraging.

**Table 4:** Estimated δ^13^C and δ ^15^N values of white shark focal areas.

	δ^13^C		δ^15^N	
Focal area	Mean	SD	Mean	SD
California	−15.8	0.6	17.3	1.4
Pelagic	−18.6	0.4	12.3	1.9
Hawaii	−17.1	0.4	11.9	0.6

**Figure 5:**
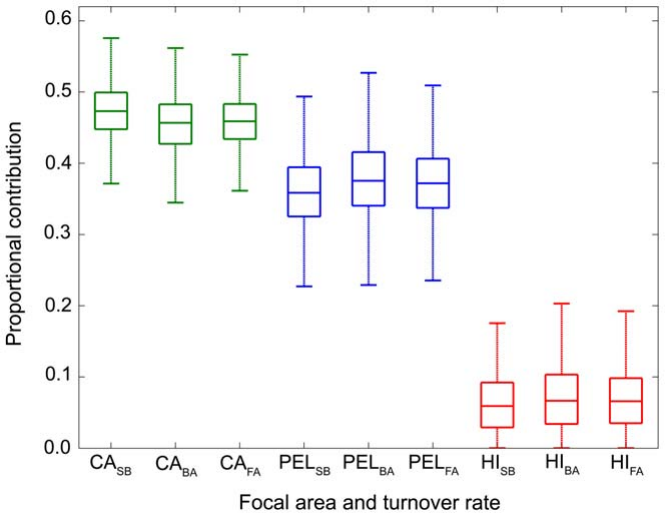
Overall contribution of different focal areas to white shark tissues estimated using the spatially explicit Bayesian mixing model. Results show posterior model estimates (median, interquartile range and max/min values) of source (CA: California, PEL: Pelagic, HI: Hawaii) contribution to muscle. The estimated contribution of the different focal areas incorporates electronic tag data from Jorgensen et al. [4] and is estimated for three different tissue incorporation rates (SB: juvenile sandbar shark, BA: allometrically scaled bird, FA: allometrically scaled fish, see text for details).

**Figure 6:**
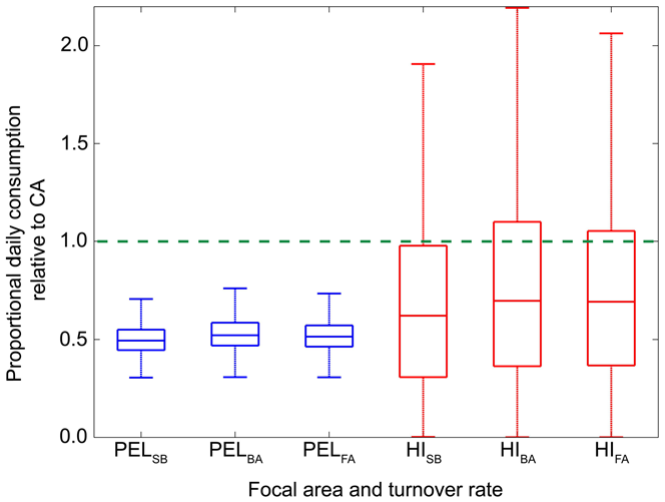
Rate of consumption in offshore focal areas (PEL: Pelagic, HI: Hawaii) relative to California (CA) estimated using the spatially explicit Bayesian mixing model. Results show posterior model estimates (median, interquartile range and max/min values). The green dashed line designates the relative consumption rate in the California focal area. Consumption rate is estimated using three different tissue incorporation rates for both offshore focal areas (SB: juvenile sandbar shark, BA: allometrically scaled bird, FA: allometrically scaled fish, see text for details).

To ensure convergence, the modified Bayesian stable isotope mixing model was run on white muscle data to produce 100,000 posterior draws and the chain was thinned for every 10^th^ draw. Mixing model results indicate that regardless of incorporation rate, white shark muscle primarily reflects California and Pelagic sources with a lower contribution from Hawaii prey (Table 5, Figure 5). Depending on the incorporation rate, the median estimated contribution of California prey to muscle tissue ranged from 45.7 to 47.3%, Pelagic prey 35.9 to 37.5%, and Hawaii prey 5.9 to 6.7% (Table 5, Figure 5). Estimated daily consumption rates were approximately half in the Pelagic region relative to the California region (median 49.5 to 52.2%), with consumption rates in Hawaii slightly higher than the Pelagic region (62.2 to 69.3% relative to California) (Table 6, Fig. 6). The estimated daily consumption rate when in Hawaii had much higher variability relative to the Pelagic region, probably due to the relative lack of tagging data from sharks going to Hawaii and the model's inability to precisely discern the location of sharks in the distant past relative to tissue incorporation rates based on isotope data. The expected stable isotope values of white shark muscle, δ^13^C −15.8‰±0.08 and δ^15^N 17.6‰±0.2 (Muscle (est), Figure 4b), reflects expectations based upon observed migratory pattern (Figure 2) and assumed equal consumption rates in different focal areas. This assumes that the different focal areas serve the same function and are used in the same manner by white sharks, an assumption that is almost certainly invalid for this species and many migratory species which move between foraging and reproductive habitats [91]. That the observed values were different from expected values indicates that this assumption is likely incorrect and demonstrates the importance of incorporating consumption rate into the mixing model.

**Table 5:** Overall contribution of different focal areas to white shark muscle tissue using different tissue incorporation rates.

	California		Pelagic		Hawaii	
Incorporation rate	Median	95%	Median	95%	Median	95%
Sandbar shark	47.3	40.1–54.4	35.9	26.2–46.7	5.9	0.3–15.3
Allometric bird	45.7	38.1–52.8	37.5	27.8–48.9	6.7	0.4–17.7
Allometric fish	45.9	38.7–53.2	37.2	27.8–49.4	6.6	0.6–16.6

Estimates incorporate tissue incorporation rate, satellite tag data, and consumption rate. Median and 95% credible intervals are presented.

**Table 6:** Estimated rate of consumption in offshore focal areas relative to consumption rate in California using different tissue incorporation rates.

	Pelagic		Hawaii	
Incorporation rate	Median	95%	Median	95%
Sandbar shark	49.5	35.7–66.7	62.2	3.0–162.9
Allometric bird	52.5	37.3–72.3	69.8	3.9–191.9
Allometric fish	51.5	37.6–71.8	69.4	6.0–178.2

Median and 95% credible intervals are presented.

## Discussion

Over the past decade, electronic tagging data have shown that a variety of large marine predators, including fish, sharks, pinnipeds, whales, marine reptiles, and marine birds, exhibit seasonally recurring migrations in the northeastern Pacific, often between coastal and oceanic habitats [92]. Seasonal migrations often take animals between areas used for foraging and ones used for reproduction [91], so understanding how a migratory species uses different parts of its range is fundamental to understanding its ecology and life history. Because productive coastal habitats and oligotrophic offshore habitats of the NEP have very different stable isotope baseline values [30], the opportunity exists to use stable isotope analysis to investigate the relative importance of different regions as foraging habitats for white sharks, and thereby shed light on the underlying function of white shark migrations.

In this study we developed a novel approach to directly integrate movement data and tissue incorporation rates into stable isotope mixing models to investigate the trophic ecology of a highly migratory marine predator, the white shark, in order to better understand how the species uses different focal areas and elucidate the life history function of its migratory behavior. Mixing model results show considerable dietary inputs from offshore pelagic habitats, in particular oceanic regions like the Café, and provide direct evidence of offshore foraging in NEP white sharks. Estimated rates of daily consumption indicate that although white sharks forage while offshore, they do so at a lower rate in offshore habitats relative to coastal ones. This is the first use of biogeochemical methods to estimate relative foraging rates in different food webs. These results suggest that white sharks may seasonally play an important role in offshore ecosystems of the NEP and provide insight of the underlying function of white shark migratory behavior.

### Evidence of offshore foraging

Although the resolution of stable isotope data generally precludes identification of specific prey species, the results of this study indicate that white sharks are foraging in offshore food webs that are depleted in ^13^C and ^15^N relative to marine mammals in California. Mixing model results indicate that while offshore, white sharks forage more on prey that are isotopically similar to the Pelagic prey rather than Hawaiian prey, indicating increased foraging in the Pelagic region relative to the Hawaiian Region. These results are consistent with the results from previous studies, which show white sharks have higher occupancy of the Café waters relative to Hawaiian waters [2], [3], [4]. Furthermore, the different incorporation rates did not greatly change mixing model results and suggests that our model was not sensitive to differences in incorporation rates in the range that we used.

The reduced consumption rate in offshore focal areas relative to California is logical given the abundance and diversity of calorically-rich prey in productive coastal habitats such as the California Current. Marine mammals, pinnipeds in particular, are especially abundant in California waters and are known to be important prey for white sharks in the region [46], [47], [48]. The oligotrophic Subtropical Gyre supports a less abundant and diverse prey field that is more patchily distributed, so there are fewer opportunities to forage, especially upon calorically-rich marine mammals. The increased consumption rate in Hawaii relative to the Pelagic region suggests that the Hawaiian region, and neritic habitats of the Hawaiian Islands in particular, may provide white sharks with increased foraging opportunities relative to the Pelagic region. This is logical given the increased abundance of prey available for white sharks to exploit in the Hawaiian Islands, including marine mammals such as monk seals and humpback whales.

A reduced rate of consumption in offshore habitats is consistent with visual observations that white sharks are often lean when they return from offshore regions to aggregation sites in central California [7]. Once sharks return to coastal aggregation sites, they rapidly gain mass by feeding on pinnipeds [7]. Hence, despite the fact that white sharks generally spend less time in coastal habitats [4], these coastal regions may play a critical role in the life history of large white sharks as foraging areas. White sharks also demonstrate a high degree of inter- and intra-annual fidelity to coastal aggregation sites, which indicates the important role of these habitats in the life history of the NEP white shark population [3], [4]. Marine mammal consumption in coastal habitats may play an important role in generating the caloric reserves that are used by sharks over the course of their offshore migrations.

Despite the likely reduction in foraging opportunities in the Subtropical Gyre, white sharks are offshore during a period of time when prey availability in this region may be relatively high. It is logical that white sharks would time their offshore migrations to coincide with periods of increased prey availability. The reported spawning area of the neon flying squid (*Ommastrephes bartrami*) generally overlaps temporally and spatially with white sharks in offshore waters [16] as does that of the purpleback flying squid (*Sthenoteuthis oualaniensis*) [17], suggesting that spawning aggregations of Ommastrephid squid may be an important prey resource in offshore waters. Similar to the neon flying squid, Pacific pomfret (*Brama japonica*) also make seasonal migrations, foraging in the transition zone or subarctic waters during the summer then moving south to subtropical waters to spawn during winter and spring [93], [94]. Catches of pelagic fishes and sharks by the Japanese long line fishery can be substantial, though not necessarily at their peak, in this region during the time of year that white sharks are present [13], [14]. It has also been noted that the occurrence of white sharks in Hawaii coincides with birthing of humpback whales [26]. Therefore, it appears that white sharks may be using these offshore habitats when prey availability, though likely still lower than in coastal habitats, is relatively greater than at other times of the year.

Although these offshore habitats may not provide optimal foraging conditions at present, it is important to remember that historical conditions may have been very different. It is possible that with the decline of large pelagic fishes, sharks and whales [5], [95], [96] the historical forage base of white sharks in these habitats has diminished. In addition, the decline of the Hawaiian monk seals [97], may have also removed an important prey item for white sharks. It seems likely that historically there was a more abundant prey field available for white sharks to exploit in pelagic habitats. Therefore, to some extent white shark migrations may be vestigial and reflect historical conditions.

There are no empirical data on white shark diet in NEP offshore waters; however, the list of potential white shark prey represents a reasonable first estimate of white shark prey in the offshore focal areas. Despite possible biases of the offshore regional estimates and potential limitations in our ability to differentiate between use of the Pelagic and Hawaiian regions, our estimates of the relative contribution of the California region vs. offshore regions (Pelagic and Hawaii) are useful because they clearly demonstrate considerable offshore foraging. It is also important to note that our data were primarily from male sharks, so our inferences largely pertain to males.

### Shift in muscle isotopic composition with size

The decrease in muscle δ^13^C and δ^15^N values with shark length is the opposite of what would be expected with ontogenetic increases in trophic level, which would lead to increased δ^13^C and δ^15^N values, and may instead be indicative of ontogenetic changes in habitat use. There is an ontogenetic shift in white shark diet with smaller sharks (<300 cm) primarily being piscivorous and larger sharks primarily consuming marine mammals [21], [98]. Therefore, we would predict that δ^13^C and δ^15^N values would increase with size as sharks consume larger, higher trophic level prey, especially marine mammals, as has been noted in Atlantic white sharks [64]. However, the opposite trend was found. The highest muscle δ^13^C and δ^15^N values in this study population were the two smallest sharks (2.74 and 3.20 m TL) and larger sharks being more depleted in ^13^C and ^15^N.

This result suggests that the smallest sharks were residential within the coastal waters of the California Current and foraged primarily in the productive coastal food webs that are enriched in ^13^C and ^15^N. Smaller juveniles (<2.5 m) are known to be coastal residents that utilize the neritic habitats of the Southern California Bight and Baja California, Mexico [25], [99]. Interestingly, two sharks (320 and 335 cm TL), which were slightly larger than the smallest two sharks, had lower isotopic values and more resembled larger sharks. In particular, the δ^15^N values of these sharks were dramatically lower. One explanation is that these two sharks specialized on lower trophic level prey (hence lower δ^13^C and δ^15^N values) than the two smallest sharks. However there is currently no evidence of such dietary specialization in white sharks. Alternatively, the decline in δ^13^C and δ^15^N in these sharks may be attributed to a threshold age at which white sharks begin offshore migrations and consumption of offshore prey. Patterns of movement and habitat use in these intermediate size white sharks (∼2.5 to 3.5+ m) are poorly understood as relatively few sharks in this range have been tagged and tracked [2], [3], [4]. The size at which white sharks begin to migrate to offshore regions is unknown and may differ with gender. Based on the similarity of the isotopic values between these two small sharks and larger sharks, for which movement patterns are well understood, the most parsimonious explanation for the δ^13^C and δ^15^N shift in smaller size classes of sharks is due to a shift in foraging habitat (onshore vs. offshore) rather than dietary differences in coastal waters.

If this shift in δ^13^C and δ^15^N values with size is due to a change in habitat use, it suggests that white sharks may start using offshore waters at lengths of 3 to 4 m (∼5 to 9 years) [87], [88], [100], a size range that encompasses the estimated size of first maturity in males (3.8 m [87]). Our results are supported by Kerr et al. [101] who reported the same decrease in δ^13^C values with white shark size based on SIA of NEP white shark vertebrae. The decrease Kerr et al. [101] reported in δ^13^C values appears to have started at similar size/age as we observed here. Kerr et al. [101] also determined that radiocarbon (^14^C) values were depleted in larger sharks. They attributed this decrease in δ^13^C and radiocarbon to ontogenetic shifts in foraging location, including increased foraging in offshore, deep, radiocarbon depleted waters [35], [102], [103]. Our sample size in this smaller size range is limited, so additional sampling of smaller sharks (<4 m) is needed and would provide insight to the timing of ontogenetic shifts in white shark habitat use.

Our observation of decreasing muscle δ^13^C and δ^15^N values with shark size may also reflect an allometric shift in incorporation rate [77], [104], with larger sharks having a slower incorporation rate. If this is the case, shark tissue would be expected to increasingly reflect offshore prey as sharks get larger since they spend more time in offshore (mean 234 d±45 SD) rather than coastal habitats. Alternatively, it is possible that this pattern is due to larger white sharks spending increasingly more time in offshore habitats relative to smaller sharks. However, currently there are not enough data to test this.

### Purpose of white shark migrations

One of the fundamental questions regarding white sharks in the NEP is why they undertake these extensive and regular offshore migrations, leaving the highly productive California Current for oligotrophic waters of the central Pacific. The two primary hypotheses are that these movements are related to foraging or reproduction. While our data are not able to directly address the reproductive hypothesis, they are useful for evaluating the foraging hypothesis. Our results indicate that 1) white sharks do forage in offshore habitats, though at a lower rate, and 2) white sharks may initiate these offshore migrations around the size of first maturity. These results, in combination with the observation that white sharks returning to central California aggregation sites often are lean [7], suggest that although white sharks feed offshore, it appears foraging may not be the primary motivation for offshore migration.

If white sharks are not moving offshore to feed, an alternative explanation is that the offshore migrations have a reproductive purpose, possibly playing a role in gestation, parturition or mating [2], [3], [4]. The particular reproductive function that offshore habitats may play in white shark life history is unclear, but it is possible that use of these habitats is related to gestation and/or mating as parturition is believed to occur in the southern California Bight [24]. It is possible that females use the warm waters of the Subtropical Gyre to aid in gestation [105], [106], and indeed there is some evidence that females segregate from males in offshore habitats, especially when in the Hawaiian focal area [107], [108]. If female white sharks have an extended gestation period (12 to 18 months [109]), extended use of warm offshore waters may explain the return of some large females to coastal aggregation sites every other year [110], [111].

Weng et al. [2] and Jorgensen et al. [4] noted that when males were in the Café they exhibited a very unusual and distinct rapid oscillatory diving behavior. Weng et al. [2] noted that this unique behavior was similar to the oscillatory diving behavior recorded by electronic tags during the spawning of Atlantic bluefin tuna in the Gulf of Mexico [112], and suggested that these behaviors may represent some sort of courtship or other reproductive behavior. Furthermore, Jorgensen et al. [4] suggested that it was possible that mating occurred offshore based on the pattern of distribution, estimated gestation time and timing of parturition, and behavior of sharks when offshore. It is unclear why sharks would not mate when both sexes are gathered at coastal aggregation sites, but several decades of visual observations at these coastal sites have yielded no evidence of mating behavior [3], [4], [110].

Because our samples are primarily from males, it is possible that the reduced offshore consumption rate that we observed is a reflection of the males engaging in periods of courtship or reproductive behavior, possibly including rapid oscillatory diving behavior, at the expense of time spent foraging. As Jacoby et al. [108] noted, it is possible that sex-specific differences in life-history traits may result in female sharks that allocate more time and energy to finding environmental conditions that aid in gestation whereas male sharks primarily focus on the pursuit of mates. Although we only had two muscle samples from females, they were indistinguishable from males, suggesting that females may also have a reduced rate of consumption. However, our ability to draw inferences for females is limited by low sample size, and possible differences in time spent offshore relative to males would need to be taken into account [110], [111].

In addition to a reduced foraging rate in offshore habitats, initiation of offshore migration around the age of first maturity is also consistent with a reproductive function to the migration. Our results suggest that sharks appeared to exhibit a shift to offshore prey between 3–4 m, the size range that encompasses the estimated size of first maturity for males. It is possible that there is no impetus for smaller, immature sharks to leave the productive nearshore habitats until there is a reproductive imperative. Our sample size is low in these smaller size ranges, so these inferences are speculative and require more research. It is possible that given the dietary shift that occurs around 3 m, when sharks start to consume marine mammals, that some shift in trophic ecology plays a role as well.

Although based on our data and modeling framework foraging does not appear to be the primary driver of offshore migration, it is likely an important component given the amount of time white sharks spend offshore. Importantly, the foraging and reproduction hypotheses are not mutually exclusive and these migrations are likely driven by a suite of factors, including 1) the physiological and reproductive condition of the sharks and 2) environmental and prey dynamics in offshore and coastal habitats. Environmental conditions or prey availability in the coastal areas may play a role as well. Jorgensen et al. [4] noted that the offshore phase coincides with the period of peak upwelling of cold water in the California Current, so it is possible that white sharks, or their prey, may have some physiological thermal limitations, leading to a reduction in use of the California Current during cold periods. Additionally, recent electronic tagging work has shown that timing of offshore migration in white sharks is coincident with offshore movements of a number of upper trophic level species from California Current, including pinnipeds, sharks, and tuna [92], suggesting a possible decrease in prey base on coastal habitats.

Regardless of the function of offshore migration, our results show that sharks clearly feed when offshore, although at a lower rate than in coastal habitats. These results do not provide support for foraging being the *primary* purpose of offshore migrations, and therefore suggest an alternative purpose such as reproduction. However, whether it is indeed reproduction or some other factor or suite of factors remains to be demonstrated.

### Dermal tissue

The dermis was highly enriched in ^13^C and ^15^N relative to muscle tissue and more closely resembled values of marine mammals in California. The mean dermis δ^13^C and δ^15^N value, corrected to resemble muscle (Dermis (corr), Figure 4b, mean δ^13^C and δ^15^N −14.9‰ and 21.5‰, respectively), is very similar to what would be expected for a diet composed of elephant seals, sea lions, and northern fur seals (prey items 4, 5 and 7, Figure 4). Elephant seals and sea lions are believed to be the primary prey of white sharks at coastal aggregation sites in central California [46], [48]. These results are consistent with a more rapid incorporation rate in dermis than muscle, thus dermis may represent more recent foraging while muscle represents a longer integration of foraging and movement. However, it is possible that isotopic routing [68], [113] or differences in amino acid composition may account for this difference.

The stable isotope dynamics of dermis have not been described for elasmobranchs or teleosts. Studies featuring human subjects show that dermal collagen, the primary component of dermis in mammals as well as elasmobranchs, is a dynamic tissue that responds rapidly to physical activity [114] and has a incorporation rate similar to or potentially faster than skeletal muscle [115], [116]. In addition to serving as a protective sheath, shark dermal collagen is believed to serve a biomechanical function where it serves as an anchorage for the body musculature [89]. Thus, dermal collagen could be subject to relatively high rates of mechanical stress and could have an increased incorporation rate. This is speculative, and experimental work is needed to assess the dynamics of isotopic incorporation of dermal collagen in elasmobranchs. If dermal collagen does incorporate dietary carbon and nitrogen more rapidly than muscle, it would be a valuable tissue for SIA because other tissues with increased incorporation rates can only be sampled destructively (e.g., liver) or require extensive handling (e.g., blood). If dermal tissue has an increased tissue incorporation rate relative to muscle, researchers could easily and non-destructively sample two tissues that provide an estimate of recent and longer term patterns of foraging and movement.

### Future directions

Given the low cost of SIA and existence of electronic tag data sets for a variety of species, the approach of integrating movement data and SIA described in this paper has the potential to be used on a variety of species and may prove useful as a monitoring tool. This approach can be used to monitor long-term patterns in trophic ecology and movements of white sharks or other marine organisms relative to fluctuations in prey availability or oceanographic conditions. NEP white sharks are ideal organisms for this approach given their small population size and relatively well-defined migratory patterns. A long-term sampling regime using these and improved techniques to monitor NEP white sharks may provide valuable information on the influence of environmental variability in offshore and nearshore habitats on white shark behavior. These techniques could also be applied to the white shark population at Guadalupe Island, Mexico, which exhibit a similar migratory pattern as central California white sharks [3]. White sharks in New Zealand [117] and South Africa [118] have also been noted to migrate to subtropical or tropical regions and comparative analyses with eastern Pacific sharks would be valuable. Additionally, in order to begin to validate this approach, efforts should be made to biopsy individual sharks with a known migratory history. If the previous year's migratory pattern of an individual shark, recorded using an electronic tag, matches the inferred pattern of habitat use based on the stable isotope composition of its muscle, it would provide some validation that this approach is accurately recording migratory pattern.

In addition to increasing the sample size of females and juvenile sharks, future work should focus on improving the parameters used in this study. Much work needs to be done to characterize the isoscapes over which these sharks move [34], generating robust isotopic data sets of potential prey in different focal regions (especially the Café and Hawaii) and refining estimates of white shark incorporation rates and discrimination factors. Such information will lead to a better understanding of the trophic ecology of this species and a more robust estimate of the dietary contribution of the different focal areas than is currently possible. Determining a better estimate of tissue incorporation rates that include body size, growth rate, and metabolic turnover of tissue for white sharks would be ideal; however the size and handling difficulty of adult white sharks makes it unlikely that we will be able to determine species-specific isotopic incorporation parameters and we may remain limited to using estimates from proxy species. In addition, obtaining a robust time series of samples from sharks throughout the coastal period, ranging from newly returned sharks to ones that have been foraging coastally for several months, may provide insight into tissue incorporation rates as sharks consume coastal prey in addition to providing information on use of coastal prey resources. Concurrent with the refinement of these parameters, use of compound-specific isotopic analysis would provide great insight into the ecology of white sharks, and elucidate how metabolic processes, trophic ecology, and movements across isotopic gradients influence the isotopic composition of a shark's tissues [119].

### Conclusions

Our results demonstrate how stable isotope analysis, when used in concert with electronic tagging data, can provide valuable insight into the ecology of white sharks in the NEP. The stable isotope composition of white shark tissues serves as a natural tracer that provides a record of their use of different regions of the NEP. The isotopic composition of white shark muscle provides strong evidence of offshore foraging, especially in pelagic habitats like the Café, by NEP white sharks, whereas dermal tissue appears to reflect recent foraging on prey in the California Current. Our results indicate that when in oligotrophic offshore waters, white sharks feed at approximately half the rate they do when in productive nearshore habitats. The decrease in muscle δ^13^C and δ^15^N values with shark length is likely indicative of ontogenetic changes in habitat use, possibly related to the onset of maturity. Overall our results suggest that although sharks feed when offshore, foraging is unlikely to be the primary purpose of the offshore migrations. If this is the case, it is possible that the extensive migrations serve a reproductive function, though this remains to be proven.

The results presented here are based on models with data-driven parameter estimates, and this study has produced results that are concordant with and add to previous research while generating new hypotheses that can be addressed in future research. Stable isotopes provide an inexpensive intrinsic tracer that can be used to infer movements and foraging in large numbers of animals. Consequently, SIA may be a cheap and cost effective way to further study and monitor general patterns of white shark migration and foraging ecology and be a useful tool for white shark conservation and management efforts. In addition, the techniques used in this study could easily be applied to other species, especially ones for which spatio-temporal patterns of distribution are relatively well understood.

## Supporting Information

Text S1 Expanded description of selection of prey species used to estimate mean δ^13^C and δ^15^N values for different focal areas (doc).
(DOC)

Text S2 JAGS/WinBUGS code for spatially explicit Bayesian mixing model. This file includes descriptions of the types and formatting of data needed to run this code (R).
(R) Click here for additional data file.
