# Supplementary material for: Using Stable Isotope Analysis to Understand the Migration and Trophic Ecology of Northeastern Pacific White Sharks (Carcharodon carcharias)
Source: PLoS One. 2012 Feb 15;7(2):e30492. doi: 10.1371/journal.pone.0030492 (PMC3280240; doi:10.1371/journal.pone.0030492)
Supplement: Text S1 — Expanded description of selection of prey species used to estimate mean δ13C and δ15N values for different focal areas (doc). (DOC) [file pone.0030492.s001.doc]

A literature review was used to obtain stable isotope values for all known and potential white shark prey in the ENP, and potential prey were grouped according to the three defined regions in order to isotopically characterize the different focal areas (Table 1). By including a diversity of potential prey, the variability associated with our regional estimates increased and provided a more conservative estimate of the mean regional value. If isotope data for a particular species and region were available from multiple studies, the study that had the largest sample size was used. A prey species’ isotopic values are assumed to be representative of both that species and taxa with similar trophic ecologies (e.g. tuna represent upper trophic level pelagic fish) and habitats. Although there are isotopic studies of potential white shark prey, many use different tissue substrates (muscle, bone collagen, tooth dentin), which may have different isotopic values based on metabolic fractionation processes or tissue incorporation rates [1,2,3]. To account for differences in discrimination between muscle and collagen (bone and tooth dentin) in marine mammals, the only prey for which collagen values were reported, we adjusted collagen δ^13^C values to resemble muscle values [4]. Because collagen turns over at a much slower rate than muscle, often integrating diet over years [3] this correction assumes that the long term diet recorded by bone is comparable to the shorter term diet recorded by muscle. Nitrogen stable isotope values were not adjusted because there is little variation in nitrogen fractionation between muscle and collagen in mammals [4,5].

The diet of white sharks in coastal California has been qualitatively described and there are a relatively high number of prey species from the region that have been characterized isotopically; therefore, the California regional estimate is likely robust in its categorization of white shark prey. Central California white sharks are believed to feed extensively on marine mammals (especially pinnipeds) [6,7,8,9]. Only isotope values from marine mammals that were sampled in the neritic waters of California were used as the California group (Table 1) [10,11,12,13,14,15].

There is a lack of basic information regarding white shark foraging ecology when offshore. In addition, there are few published stable isotope data for potential prey species in offshore habitats and no samples have been collected directly within the Café. However, the Café is part of the eastern subtropical gyre; thus species sampled from nearby regions of this large oceanographic region that have similar oceanographic conditions and biogeochemical processes [16,17,18,19,20] should be isotopically similar to those species in the Café. In addition, potential prey of white sharks in offshore habitats includes species that are not residents of the Café or Hawaii but occur seasonally in these regions and therefore might be encountered by white sharks in offshore habitats. As a result, stable isotope values from organisms sampled in different regions of the ENP were used to define the Pelagic region, which generally covers offshore, pelagic habitats between Hawaii and California, and serves as our proxy for the Café and similar habitats.

Several potential prey items in the Pelagic group were caught in the California Current System, but due to known migratory patterns could be encountered by white sharks when offshore. Blue shark (*Prionace glauca*) values from the west coast of the U.S. (Robert Leaf, Virginia Tech, unpublished data) were used because this species is a widely distributed and highly migratory species [21,22,23] that is known to occur in the Café and be consumed by white sharks [24] making these values our best estimate for a pelagic shark species that white sharks may encounter during offshore periods. Similarly, albacore (*Thunnus alalunga*) and bluefin tuna (*Thunnus orientalis*) values (Daniel J. Madigan, unpublished data) are from fish caught in the California Current, but electronic tag and isotopic data suggest that these values are representative of fish that foraged in offshore pelagic habitats away from California prior to being caught in California.

Ommastrephid squid used in the Pelagic region are from areas to the north of Hawaii, as far north as 45°N [25,26,27], but the isotopic composition of these squid is likely representative of the squid that white sharks may encounter in the Café. These squid either forage at higher latitudes along the North Pacific transition zone [28] and migrate south to spawn in waters in the subtropical gyre during the period when white sharks are present (neon flying squid *O. bartramii*) or are resident in the subtropical gyre throughout the year and spawn in the spring and summer when white sharks are present (purpleback flying squid *S. oualaniensis*) [29,30,31,32]. The Pacific pomfret, sampled to the north of Hawaii, is one of the dominant large pelagic fishes in the ENP and was used as a representative mid- to upper trophic level pelagic fish, and like the neon flying squid makes seasonal migrations from the transition zone or subarctic waters to subtropical waters to spawn during winter and spring [33,34]. We used the northern right whale dolphin, sampled to the north of Hawaii, as a representative of small mid- to upper-trophic level cetaceans in offshore pelagic habitats.

This species is abundant in offshore waters of the North Pacific [35], and was the most frequently killed small cetacean in the high-seas driftnet fishery in the North Pacific [36]. Although the Café is at the southern extent of their range, their abundance throughout the North Pacific suggests that white sharks may encounter them when offshore.

Data for the Hawaii region are from the nearshore waters of the Hawaiian Islands as well as pelagic waters to the south [37,38,39]. The Hawaii value comprised four species: bigeye tuna (*Thunnus obesus*), yellowfin tuna (*Thunnus albacares*), purpleback flying squid (*Sthenoteuthis oualaniensis*), and humpback whales (*Megaptera novaeangliae*). Electronic tagging studies [40,41,42] and isotopic studies [20] have shown that bigeye and yellowfin tuna in the Pacific exhibit restricted horizontal movements and display a relatively high degree of regional residency, indicating that the isotopic composition of Hawaiian tuna used in this study represent a reasonable estimate of upper trophic level pelagic fishes that white sharks may feed upon in nearshore Hawaiian waters [20], as these tuna were all caught at nearshore fish attracting devices in the main Hawaiian Islands [38]. Humpback whales sampled in coastal Hawaiian habitats feed at high latitudes in the North Pacific and migrate to Hawaii to calve [13,37]. The squid used in the Hawaii region (Douglas McCauley, Hopkins Marine Station of Stanford University, unpublished data) are from offshore pelagic habitats (approximately 6 - 7°N, south of the main Hawaiian Islands) from southern extent of the Hawaiian white shark focal area and were sampled during May, when white sharks may occur in that region [43].

**Literature cited**

1. Gannes LZ, O'Brien DM, Martinez Del Rio C (1997) Stable isotopes in animal ecology: assumptions, caveats, and a call for more laboratory experiments. Ecology 78: 1271-1276.

2. Martinez del Rio C, Wolf N, Carleton SA, Gannes LZ (2009) Isotopic ecology ten years after a call for more laboratory experiments. Biol Rev 84: 91-111.

3. Koch PL (2007) Isotopic study of the biology of modern and fossil vertebrates. In: Michener R, Lajtha K, editors. Stable isotopes in ecology and environmental science. 2nd ed. Boston: Blackwell Publishing. pp. 99 - 154.

4. Nardoto GB, Godoy PB, Ferraz ESB, Ometto JPHB, Martinelli LA (2006) Stable carbon and nitrogen isotopic fractionation between diet and swine tissues. Sci Agric 63: 579-582.

5. Roth JD, Hobson KA (2000) Stable carbon and nitrogen isotopic fractionation between diet and tissue of captive red fox: implications for dietary reconstruction. Can J Zool 78: 848-852.

6. Ainsley DG, Henderson RP, Huber HR, Boekelheide RJ, Allen SG, et al. (1985) Dynamics of white shark/pinniped interactions in the Gulf of the Farallons. Mem South Calif Acad Sci 9: 109-122.

7. Le Boeuf BJ, Riedman M, Keyes RS (1982) White shark predation on pinnipeds in California coastal waters Fish Bull 80: 891-895.

8. Long DJ, Hanni KD, Pyle P, Roletto J, Jones RE, et al. (1996) White shark predation on four pinniped species in central California waters: geographic and temporal patterns inferred from wounded carcasses. In: Klimley PA, Ainsley DG, editors. Great white sharks: the biology of *Carcharadon carcharias*. San Diego: Academic Press. pp. 263-274.

9. Long DJ, Jones RE (1996) White shark predation and scavenging on cetaceans in the eastern North Pacific Ocean. In: Klimley PA, Ainsley DG, editors. Great white sharks: the biology of *Carcharadon carcharias*. San Diego: Academic Press. pp. 293-307.

10. Jarman WM, Hobson KA, Sydeman WJ, Bacon CE, McLaren EB (1996) Influence of trophic position and feeding location on contaminant levels in the Gulf of the Farallones food web revealed by stable isotope analysis. Environ Sci Technol 30: 654 - 660.

11. Burton RK, Koch PL (1999) Isotopic tracking of foraging and long-distance migration in northeastern Pacific pinnipeds. Oecologia 119: 578-585.

12. Burton RK, Snodgrass JJ, Gifford-Gonzalez D, Guilderson T, Brown T, et al. (2001) Holocene changes in the ecology of northern fur seals: insights from stable isotopes and archaeofauna. Oecologia 128: 107-115.

13. Witteveen BH, Worthy GAJ, Wynne KM, Roth JD (2009) Population structure of North Pacific humpback whales on their feeding grounds revealed by stable carbon and nitrogen isotope ratios. Mar Ecol Prog Ser 379: 299-310.

14. Topperoff AK (1997) Diet of harbor porpoise (*P. phocoena*) using stomach contents and stable isotope analyses [MS]. San Jose: San Jose State University. 103 p.

15. Newsome SD, Etnier MA, Monson DH, Fogel ML (2009) Retrospective characterization of ontogenetic shifts in killer whale diets via δ^13^C and δ^15^N analysis of teeth. Mar Ecol Prog Ser 374: 229-242.

16. Goericke R, Fry B (1994) Variations of marine plankton δ^13^C with latitude, temperature, and dissolved CO_2_ in the world ocean. Global Biogeochemical Cycles 8: 85-90.

17. Clementz MT, Koch PL (2001) Differentiating aquatic mammal habitat and foraging ecology with stable isotopes in tooth enamel. Oecologia 129: 461 - 472.

18. Michener RH, Kaufman L (2007) Stable isotope ratios as tracers in marine food webs: an update. In: Michener R, Lajtha K, editors. Stable isotopes in ecology and environmental science. 2nd ed. Boston: Blackwell. pp. 238 - 282.

19. Montoya JP (2007) Natural abundance of ^15^N in marine planktonic ecosystems. In: Michener R, Lajtha K, editors. Stable isotopes in ecology and environmental science. 2nd ed. Boston: Blackwell. pp. 176-201.

20. Graham BS, Koch PL, Newsome SD, McMahon KW, Aurioles D (2010) Using isoscapes to trace the movements and foraging behavior of top predators in oceanic ecosystems. In: West JB, Bowen GJ, Dawson TE, Tu KP, editors. Isoscapes: understanding movement, pattern, and process on earth through isotope mapping. New York: Springer. pp. 299-318.

21. Strasburg DW (1958) Distribution, abundance and habits of pelagic sharks in the central Pacific. Fish Bull 58: 335 - 361.

22. Nakano H, Seki MP (2003) Synopsis of biological data on the blue shark *Prionace glauca* Linnaeus. Bull Fish Res Agency 6: 18-55.

23. Weng KC, Castilho PC, Morrissette JM, Landeira-Fernandez AM, Holts DB, et al. (2005) Satellite tagging and cardiac physiology reveal niche expansion in salmon sharks. Science 310: 104-106.

24. Compagno LJV (2001) Sharks of the world. An annotated and illustrated catalogue of shark species known to date. Volume 2. Bullhead, mackerel and carpet sharks (Heterodontiformes, Lamniformes and Orectolobiformes). Rome: FAO Species Catalogue for Fishery Purposes No. 1 Vol. 2. 269 p.

25. Gould P, Ostrom P, Walker W (1997) Trophic relationship of albatrosses associated with squid and large-mesh drift-net fisheries in the North Pacific Ocean. Can J Zool 75: 549 - 562.

26. Parry MP (2003) The trophic ecology of two ommastrephid squid species, *Ommastrephes bartamii* and *Sthenoteuthis oualaniensis*, in the North Pacific sub-tropical gyre [Ph.D.]. Manoa: University of Hawaii. 287 p.

27. Parry MP (2008) Trophic variation with length in two ommastrephid squids, *Ommastrephes bartramiii* and *Sthenoteuthis oualaniensis*. Mar Biol 153.

28. Polovina JJ, Howell E, Kobayashi DR, Seki MP (2001) The transition zone chlorophyll front, a dynamic global feature defining migration and forage habitat for marine resources. Prog Oceanogr 49: 469-483.

29. Ichii T, Mahapatra K, Sakai M, Okada Y (2009) Life history of the neon flying squid: effect of the oceanographic regime in the North Pacific Ocean. Mar Ecol Prog Ser 378: 1-11.

30. Bower JR, Ichii T (2005) The red flying squid (*Ommastrephes bartramii*): a review of recent research and the fishery in Japan. Fish Res 76: 39-55.

31. Zuyev G, Nigmatullin C, Chesalin M, Nesis K (2002) Main results of long-term worldwide studies on tropical nektonic oceanic squid genus *Sthenoteuthis*: an overview of Soviet investigations. Bull Mar Sci 71: 1019-1060.

32. Harman RF, Young RE (1985) The larvae of Ommastrephid squids (Cephalopods, Teuthoidea) from Hawaiian waters. Vie Milieu 35: 211-222.

33. Pearcy WC, Fisher JP, Yoklavich MM (1993) Biology of the Pacific pomfret (*Brama japonica*) in the North Pacific Ocean. Can J Fish Aquat Sci 50.

34. Watanabe H, Kubodera T, Kawahara S (2006) Summer feeding habits of the Pacific pomfret *Brama japonica* in the transitional and subarctic waters of the central North Pacific. J Fish Biol 68: 1436-1450.

35. Leatherwood S, Walker WA (1979) The northern right whale dolphin *Lissodelphis borealis* Peale in the eastern North Pacific Ocean. In: Winn HE, Olla BL, editors. Behavior of Marine Animals Volume 3: Cetaceans. New York: Plenum. pp. 85-141.

36. Hobbs RC, Jones LL (1993) Impacts of high seas driftnet fisheries on marine mammal populations in the North Pacific. Int North Pac Fish Comm Bull 53: 409-434.

37. Witteveen BH, Worthy GAJ, Roth JD (2009) Tracing migratory movements of breeding North Pacific humpback whales using stable isotope analysis. Mar Ecol Prog Ser 393: 173-183.

38. Graham BS (2007) Trophic dynamics and movements of tuna in tropical Pacific Ocean inferred from stable isotope analyses [Ph.D.]. Manoa: University of Hawaii. 237 p.

39. Graham BS, Grubbs D, Holland K, Popp BN (2007) A rapid ontogenetic shift in the diet of juvenile yellowfin tuna from Hawaii. Mar Biol 150: 647-658.

40. Sibert JR, Musyl MK, Brill RW (2003) Horizontal movements of bigeye tuna (*Thunnus obesus*) near Hawaii determined by Kalman filter analysis of archival tagging data. Fish Oceanogr 12: 141-151.

41. Adam MS, Sibert J, Itano D, Holland KN (2003) Dynamics of bigeye (*Thunnus obesus*) and yellowfin (*T. albacares*) tuna in Hawaii’s pelagic fisheries: analysis of tagging data with a bulk transfer model incorporating size-specific attrition. Fish Bull 101: 215-228.

42. Schaefer KM, Fuller DW, Block BA (2007) Movements, behavior, and habitat utilization of yellowfin tuna (*Thunnus albacares*) in the northeastern Pacific Ocean, ascertained through archival tag data. Mar Biol 152: 503-525.

43. Jorgensen SJ, Reeb CA, Chapple TK, Anderson S, Perle C, et al. (2010) Philopatry and migration of Pacific white sharks. Proc R Soc Biol Sci Ser B 277: 679-688.
